# Effects of exercise on high-fat diet–induced non-alcoholic fatty liver disease and lipid metabolism in *ApoE* knockout mice

**DOI:** 10.1186/s12986-022-00644-w

**Published:** 2022-02-16

**Authors:** Wen-Ching Huang, Jin-Wei Xu, Shiming Li, Xin Er Ng, Yu-Tang Tung

**Affiliations:** 1 Department of Exercise and Health Science, National Taipei University of Nursing and Health Sciences, Taipei, 112 Taiwan; 2 Department of Forestry, National Chung Hsing University, Taichung, 402 Taiwan; 3 Department of Food Science, Rutgers University, New Brunswick, NJ 08901 USA; 4 Graduate Institute of Metabolism and Obesity Sciences, Taipei Medical University, Taipei, 110 Taiwan; 5 Graduate Institute of Biotechnology, National Chung Hsing University, Taichung, 402 Taiwan; 6 Cell Physiology and Molecular Image Research Center, Wan Fang Hospital, Taipei Medical University, Taipei, 116 Taiwan; 7 Nutrition Research Center, Taipei Medical University Hospital, Taipei, 110 Taiwan

**Keywords:** Exercise, Non-alcoholic fatty liver disease, High-fat diet, Lipidomic, *ApoE* knockout

## Abstract

**Background:**

Non-alcoholic fatty liver disease (NAFLD), which is growing more common in the Western world, has become the main cause of chronic liver disease and is strongly associated with metabolism syndromes. NAFLD can indicate a wide spectrum of hepatic pathologies, ranging from simple hepatic steatosis and inflammatory non-alcoholic steatohepatitis to more severe stages of fibrosis and cirrhosis. Moreover, evidence has demonstrated that physical inactivity and westernized dietary habits may facilitate the development of NAFLD. Lipid modulation and metabolism could be important factors in the development of steatosis. Lipid species, characterized using a lipidomic approach with untargeted analysis, could provide potential biomarkers for the pathogenesis of NAFLD or therapeutic applications. Thus, in this study, the effects of exercise on the improvement of NAFLD were further investigated from a lipidomic perspective through the aspects of lipid regulation and metabolism.

**Methods:**

Wild type (WT) C57BL/6 J and C57BL/6-*ApoE*^*em1Narl*^/Narl mice were assigned to one of four groups: WT mice fed a normal chow diet (CD), apolipoprotein E (*ApoE*) knockout mice fed a normal CD, *ApoE* knockout mice fed a high-fat diet (HFD), and *ApoE* knockout mice fed a HFD and provided with swimming exercise. The treatments (e.g., normal diet, HFD, and exercise) were provided for 12 consecutive weeks before the growth curves, biochemistry, fat composition, pathological syndromes, and lipid profiles were determined.

**Results:**

Exercise significantly reduced the HFD-induced obesity (weight and fat composition), adipocyte hypertrophy, liver lipid accumulation, and pathological steatosis. In addition, exercise ameliorated HFD-induced steatosis in the process of NAFLD. The lipidomic analysis revealed that the changes in plasma triglyceride (14:0/16:0/22:2), phosphatidic acid (18:0/17:2), and phosphatidylglycerol (16:0/20:2) induced by the administration of the HFD could be reversed significantly by exercise.

**Conclusions:**

The 12-week regular exercise intervention significantly alleviated HFD-induced NAFLD through modulation of specific lipid species in plasma. This finding could elucidate the lipids effects behind the hepatic pathogenesis with exercise.

## Introduction

Non-alcoholic fatty liver disease (NAFLD) has become a major cause of hepatic dysfunction worldwide. The average global prevalence of NAFLD is approximately 25%; however, this rate varies widely (e.g., 21%–24.7% in the USA, 12.5%–38% in Mainland China, 12%–51% in Taiwan, 23%–26% in Japan, 27% in Korea, and 9%–32% in India) due to differences in the diagnostic methods and criteria [1]. A survey conducted in the USA investigated the annual economic burden caused by NAFLD and estimated that the cost was as high as 103 and 188 billion USD from the direct medical care costs and societal costs, respectively [2]. NAFLD is associated with metabolic conditions and complications, such as obesity, diabetes, cardiovascular disease, gout, chronic kidney disease, polycystic ovary syndrome, hypothyroidism, hypogonadism, and obstructive sleep apnea. [3]. Owing to the rate of hepatic cirrhosis caused by NAFLD, it is estimated that the demand for liver transplantation will increase by 2030 [4].

The lipids stored in the liver are derived from three major sources: free fatty acids from blood, de novo lipogenesis, and dietary intake. The metabolic processes resulting from a high-fat diet (HFD) can cause oxidative stress in mitochondria and the endoplasmic reticulum, as well as induce de novo lipogenesis and inflammation. This process accelerates the development of NAFLD [5]. NAFLD includes a wide spectrum of diseases, such as simple steatosis (NAFL), non-alcoholic steatohepatitis (NASH), fibrosis, and cirrhosis. In some cases, these conditions may eventually progress into hepatocellular carcinoma [6]. NAFLD involves complex processes and underlying mechanisms, including the malfunction of mitochondria and the endoplasmic reticulum, activation of the immune system, hepatocyte apoptosis and necrosis, the deposition of iron, and dysbiosis of microbiota [7]. The lipotoxicity, excessive fat accumulation-caused malfunctions in several metabolic pathways, demonstrated insulin resistance associated with elevated circulating levels of lipids and the metabolic alterations in fatty acid utilization and intracellular signaling. The protein kinase C and the JNK-1 pathways were possibly involved as mechanisms for lipotoxicity-induced insulin resistance in nonadipose tissue organs, such as liver and muscle [8].

In therapeutic strategies for NAFLD, nutrition management (e.g., calorie limitation or the Mediterranean diet) can mitigate or prevent the progression of NAFLD [9]. The utilization of pharmaceuticals for weight loss or hyperlipidemia could also be considered by physicians for treating obesity and NAFLD. In addition, bariatric surgery may reverse hepatic pathogenesis in patients with NAFLD and NASH. However, there is a need for large randomized clinical trials to examine the beneficial effects of this surgical intervention [10]. A sedentary lifestyle, physical inactivity, and excessive caloric intake can contribute synergistically to NAFLD. Therefore, as shown by clinical evidence, lifestyle modification could be used as a primary therapy to manage NAFLD and NASH [11]. Exercise reduces the accumulation of hepatic fat with improvements in insulin resistance, liver fatty acid metabolism, and inflammation through regulation of lipogenesis genes and mitochondrial function for the amelioration of fatty liver disease [12]. Moreover, the levels of reactive oxygen species and oxidative stress in NAFLD can be suppressed by exercise, which upregulates several antioxidant enzymes and anti-inflammatory mediators. Therefore, exercise itself may prevent the progression of NAFLD [13].

Lipidomics analysis, based on the principles of analytical chemistry and mass spectrometry (MS) technology, reveals the complex lipid species related to barriers, membrane matrices, signaling, and energy depots for essential cellular functions. The MS-based techniques utilized in lipidomics analysis include liquid chromatography (LC)-based methods, shotgun, and MS imaging with different types of LC–MS, tandem MS, matrix-assisted laser desorption ionization MS, tandem MS, desorption electrospray ionization MS, and ion mobility MS. These methods are characterized by different advantages and limitations [14]. From the perspective of the lipidome, lipid metabolism may play role important roles in metabolic syndrome-related diseases and lead to the identification of modulators or biomarkers [15]. The varying lipid profiles derived from different interventions, such as nutrition, medicine, and exercise, could also provide a better understanding of disease development. Numerous studies have demonstrated that exercise can improve NAFLD and obesity. However, the lipid profile following exercise remains poorly understood. Therefore, we hypothesized that regular exercise could alleviate NAFLD induced by a HFD provided to apolipoprotein E (*ApoE*) knockout mice, possibly through the regulation of adipogenic genes and changes in the lipidomic profile.

## Materials and methods

### Animals

Male C57BL/6-*Apoe*^*em1Narl*^/Narl mice with the knockout of *ApoE* inducing spontaneous hyperlipidemia syndromes (*ApoE* knockout) and C57BL/6J (wild type [WT]) mice of the same age (seven weeks) and gender were purchased from the National Laboratory Animal Center (Taipei, Taiwan) and maintained in the laboratory animal center of Taipei Medical University under stable conditions: constant temperature (24 ± 2 °C); humidity (50 ± 5%); and circadian rhythm (12:12 h light:dark cycle). The study was approved by the Institutional Animal Care and Use Committee of Taipei Medical University, and the procedures conformed to the guidelines of LAC-2017-0230 for animal welfare. The mice were assigned to four groups (six mice per group) as follows: WT mice fed a normal chow diet (CD) (i.e., WT CD); *ApoE* knockout mice fed a normal CD (EKO CD); *ApoE* knockout mice fed a HFD (EKO HD); and *ApoE* knockout mice fed a HFD and provided with swimming exercise (EKO HD EX). Standard CD (Laboratory Rodent Diet 5001; LabDiet, St. Louis, MO, USA) with an energy density of 3.10 kcal/g (28.5% energy derived from proteins, 13.5% from fats (ether extract), and 58% from carbohydrates) and a HFD (Diet #D12079B; Research Diets, New Brunswick, NJ, USA) with an energy density of 4.67 kcal/g (17% energy derived from proteins, 40% from fats (butter and corn oil), and 43% from carbohydrates) were provided in this study for 12 weeks as dietary interventions after one week of acclimation. The body weight and dietary intake were regularly recorded during the study for the calculation of calories, the feed conversion ratio (FCR; %; net weight production/total feed weight), and the construction of growth curves. Tissues (e.g., white adipocyte tissue and liver) and blood were collected and preserved in liquid nitrogen for further analysis immediately after the study ended. Veterinarians monitored animal health status daily based on behavior and appearance and inspected the manipulation procedures to ensure animal welfare.

### Exercise protocol

The swimming exercise training was performed in a cylindrical tank without a weight load, and the water temperature was maintained at 36 °C. The mice in the EKO HD EX group practiced the swimming behavior (10 min/day) for three days prior to the exercise intervention. The duration of swimming was 30 min/day for weeks 1–2 and 40 min/day for weeks 3–12 at a frequency of five days/week. An air pump system was incorporated into the tank to produce turbulent motion from bubbles to prevent floating or resting behavior. After swimming, the trained mice were towel-dried and inspected before being returned to their cage. The exercise protocol was previously described [16], and involved slight modifications for animal physical fitness and environmental factors.

### Blood biochemical testing

After euthanasia through asphyxiation with CO_2_ (30% of the cage volume/minute replacement), blood was immediately collected by cardiac puncture in a 1.5 mL anticoagulant tube. Subsequently, the samples were centrifuged at 3,000 rpm and 4 °C for 10 min to collect plasma. The levels of total cholesterol (TC), triglyceride (TG), low-density lipoprotein cholesterol (LDL-c), high-density lipoprotein cholesterol (HDL-c), aspartate aminotransferase (AST), and alanine aminotransferase (ALT) in plasma were measured using a Roche Modular P800 device (Roche Diagnostics, Indianapolis, IN, USA).

### Histopathological analysis of adipocyte distribution and hepatic steatosis

White adipose tissues (epididymal and perirenal white adipose tissues [eWAT and pWAT, respectively]) and the liver were precisely excised and weighed to determine the body composition of the mice. Subsequently, tissues were preserved in 4% formaldehyde and embedded in paraffin. The paraffin-embedded tissue samples were sectioned (thickness: 4 μm) and stained with hematoxylin and eosin for histological observation. Each section contained five fields (approximately 150 adipocytes) of eWAT, which were systematically analyzed to determine the adipocyte area (μm^2^) and adipocyte size distribution using Adiposoft ImageJ software (National Institutes of Health, Bethesda, Maryland, USA) [17]. In addition, the hepatic histological pathogenesis of steatosis was graded according to previous pathological standards, with slight modification [18].

### Hepatic TG assessment

Hepatic TGs were assessed using an enzyme-linked immunosorbent assay colorimetric assay kit (#10010303; Cayman Chemical, Ann Arbor, MI, USA). Frozen hepatic tissue (approximately 400 mg) was homogenized with diluted NP40 substitute reagent (2 mL) and centrifuged at 10,000 g and 4 ℃ for 10 min. The supernatant was further diluted (1:10) for the assessment of TGs according to the instructions provided by the manufacturer.

### Plasma lipidomic profile analysis

The Folch method was used to extract plasma lipids, and the lipids were separated from the biphasic mixture of chloroform and methanol as previous described [19] with slight modifications. Initially, methanol (5 mL) was added to plasma samples (*n* = 6; 40 µL each) collected from the four groups. This was followed by addition of CHCl_3_ (3 mL) and incubation for 1 h at room temperature with occasional vortex mixing. Next, after adding distilled water (1.25 mL), the samples were left at room temperature for 10 min to facilitate the phase separation. After centrifugation at 10,000 g and 4 ℃ for 10 min, the organic phase was aliquoted (2 mL/sample) for vacuum drying and stored at − 80 °C until further analysis.

The samples were reconstituted with isopropanol/acetonitrile/water (2:1:1; 250 μL) for ultra-performance LC-quadrupole time of flight/MS analysis performed using SYNAPT G2 QTof (Waters MS Technologies, Manchester, UK). The MS conditions in positive ionization mode detection were as follows: desolvation gas flow 900 L/h; desolvation temperature 550 °C; cone gas flow 15 L/h; source temperature 120 °C; capillary voltage 2.8 kV; cone voltage 40 V; and time of flight-MS scan range 100–2,000 m/z. The two rapidly alternating functions with different levels of collision energy could simultaneously collect full exact masses in the Waters MS acquisition mode. All analyses were performed using LockSpray (Waters, Milford, MA, USA) to ensure accuracy and reproducibility. Leucine–enkephalin (concentration: 1 ng/μL; flow rate: 5 μL/min) was applied to the lock mass in a continuum mode. Waters MassLynx v4.1 software (Waters MS Technologies) was utilized to control the conditions for the acquisition of all data. Data were obtained from six individual samples/group, and each sample was analyzed in triplicate.

The MS data were processed using the Progenesis QI software (Waters, Milford, MA, USA) for alignment by peak picking and identification of polar lipids with high-resolution positive-ion MS. The absolute intensities of compounds identified by the Progenesis QI software were recalculated to determine the relative abundances and to normalize the values of the lipid molecules. The data were further imported into the EZinfo 2.0 software (Sartorius Stedim Biotech, Umeå, Sweden) for multivariate statistical analysis. Principal component analysis (PCA) and orthogonal projections to latent structures discriminant analysis (OPLS-DA) were used to obtain the group clusters with the final statistical models. For group clustering, lipid molecules with variable importance in the projection > 1, fold change > 1, and *p* values < 0.05 were considered to have the greatest effect.

### Statistical analysis

Data are represented as the mean ± standard deviation (SD). The statistical analysis was performed using SPSS Statistics version 22 (IBM Corporation, Armonk, NY, USA). Differences among groups in body weight, dietary, feed conversion ratio, lipemia indexes, hepatic indexes, fat composition, and adipocyte area were analyzed using one-way analysis of variance (one-way ANOVA), followed by post-hoc Duncan’s test. The lipid species profiles were analyzed by independent t test between indicated groups. A probability of a type I error < 0.05 denoted significant difference.

## Results

### Effects of exercise on growth curves, dietary intake, and FCR in mice with HFD-induced obesity

**Fig. 1:**
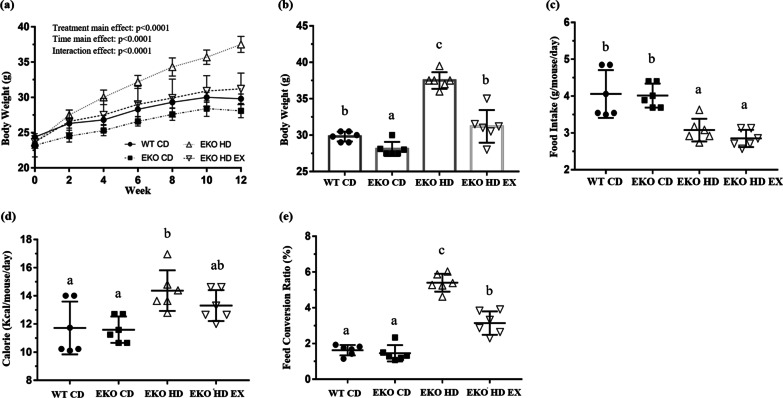
Effects of exercise on growth curve, diet, and feed conversion ratio (FCR). Related indices, such as growth curve (**a**), final body weight (**b**), food intake (**c**), caloric intake (**d**), and FCR (**e**) were recorded during the experiment. Values are presented as the mean ± SD (*n* = 6). Different letters indicate significant differences (*p* < 0.05) compared with the other groups, as assessed by one-way ANOVA. FCR (%): body weight increment/food intake × 100. EKO CD, apolipoprotein E (*ApoE*) knockout mice fed a normal chow diet; EKO HD, *ApoE* knockout mice fed a high-fat diet; EKO HD EX, *ApoE* knockout mice fed a high-fat diet along with swimming exercise; WT CD, wild type mice fed a normal chow diet

The average initial body weight in the four groups was approximately 23.7 g without significant differences (F(3,20) = 1.43, *p* = 0.265). Mice in different treatment groups were subjected to exercise and the HFD intervention for 12 weeks. Significant differences were observed in the main and interaction effects for two factors: time and treatment (F(6,120) = 557.5, *p* < 0.0001; F(3,20) = 19.7, *p* < 0.0001; F(18,120) = 31.8, *p* < 0.0001, respectively; Fig. 1a). Significant differences were observed in the simple main effects among the groups at week 12 (F(3,20) = 52.0, *p* < 0.0001). The body weight in the EKO HD group was significantly higher than that recorded in the normal CD groups (WT CD and EKO CD). The exercise intervention group (EKO HD EX) showed a significant decrease in body weight compared with the EKO HD group (Fig. 1b). The food and caloric intake also demonstrated significant differences among these groups (F(3,20) = 13.9, *p* < 0.0001 and F(3,20) = 5.54, *p* = 0.006, respectively). The food intake in the HFD groups (EKO HD and EKO HD EX groups) was significantly lower than that measured in the normal CD groups (WT CD and EKO CD groups). In accordance with the caloric intake, the EKO HD group also showed a significant increase compared with the WT CD and EKO CD groups; nevertheless, there was no significant difference between the EKO HD and EKO HD EX groups (Fig. 1c, d). The exercise training for extra energy expenditure also caused significant differences among the groups in FCR (F(3,20) = 82.9, *p* < 0.0001). The FCR of the EKO HD group showed significant increment compared with those of the normal CD groups (WT CD and EKO CD groups). In addition, the exercise intervention (EKO HD EX group) significantly ameliorated the FCR increase caused by HFD in the EKO HD group (Fig. 1e). The extra energy consumption, a result of the daily exercise training, alleviated the HFD-induced weight gain (obesity) and FCR. These potential health benefits derived from exercise may prevent the occurrence of obesity-associated complications.

### Effects of exercise on plasma biochemical parameters

**Fig. 2:**
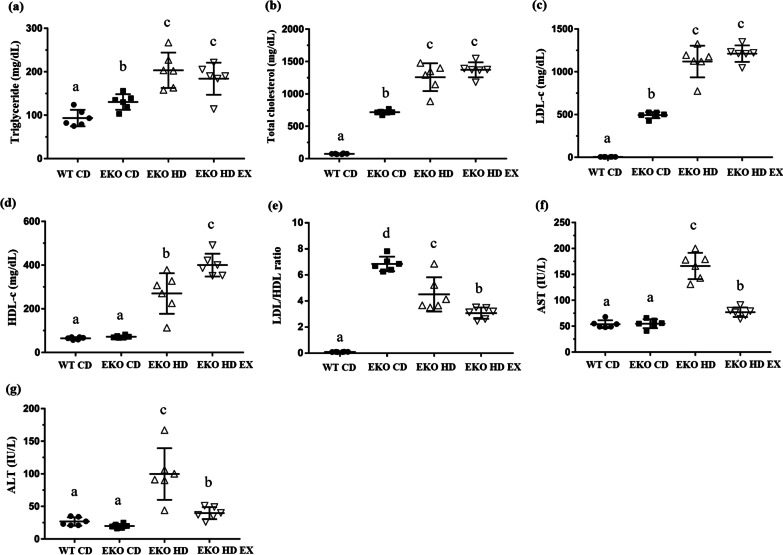
Effects of exercise on plasma biochemical variables in *ApoE* mice feed fed a high-fat diet. The levels of TG (**a**), TC (**b**), LDL-c (**c**), HDL-c (**d**), LDL/HDL (**e**), AST (**f**), and ALT (**g**) in plasma were analyzed at the end of the study. Values are presented as the mean ± SD (*n* = 6). Different letters indicate significant differences among the groups (one-way ANOVA, *p* < 0.05). ALT, alanine aminotransferase; *ApoE*, apolipoprotein E; AST, aspartate aminotransferase; HDL-c, high-density lipoprotein cholesterol; LDL-c, low-density lipoprotein cholesterol; SD, standard deviation; TC, total cholesterol; TG, triglyceride

The biochemical indices (e.g., TG, TC, LDL-c, HDL-c, AST, and ALT) reflect the physiological and health status in clinical and disease animal models. The dyslipidemia (TG, TC, LDL-c, HDL-c, and LDL/HDL ratio) and liver function (AST and ALT) indices in the current HFD-induced obesity model revealed significant differences among the groups (F(3,20) = 16.4, *p* < 0.0001; F(3,20) = 140.1, *p* < 0.0001; F(3,20) = 171.6, *p* < 0.0001; F(3,20) = 55.9, *p* < 0.0001; F(3,20) = 86.4, *p* < 0.0001; F(3,20) = 79.1, *p* < 0.0001; and F(3,20) = 18.6, *p* < 0.0001, respectively; Fig. 2). The TG, TC, LDL-c, and LDL/HDL ratio indices were elevated in the EKO CD group compared with the WT CD group due to *ApoE* knockout-associated dyslipidemia. The EKO HD group demonstrated significant increment in the TG, TC, LDL-c, HDL-c, AST, and ALT indices associated with the HFD when compared with the WT CD and EKO CD groups. The exercise intervention significantly elevated the levels of HDL-c and improved those of AST, ALT and LDL/HDL ratio in the EKO CD EX group versus the EKO HD group. However, the levels of TG, TC, and LDL-c in the EKO HD group were not significantly affected by the exercise training intervention in the present study.

### Effects of exercise on the morphology, weight, mean adipocyte size, and distribution of white adipose tissue

**Fig. 3:**
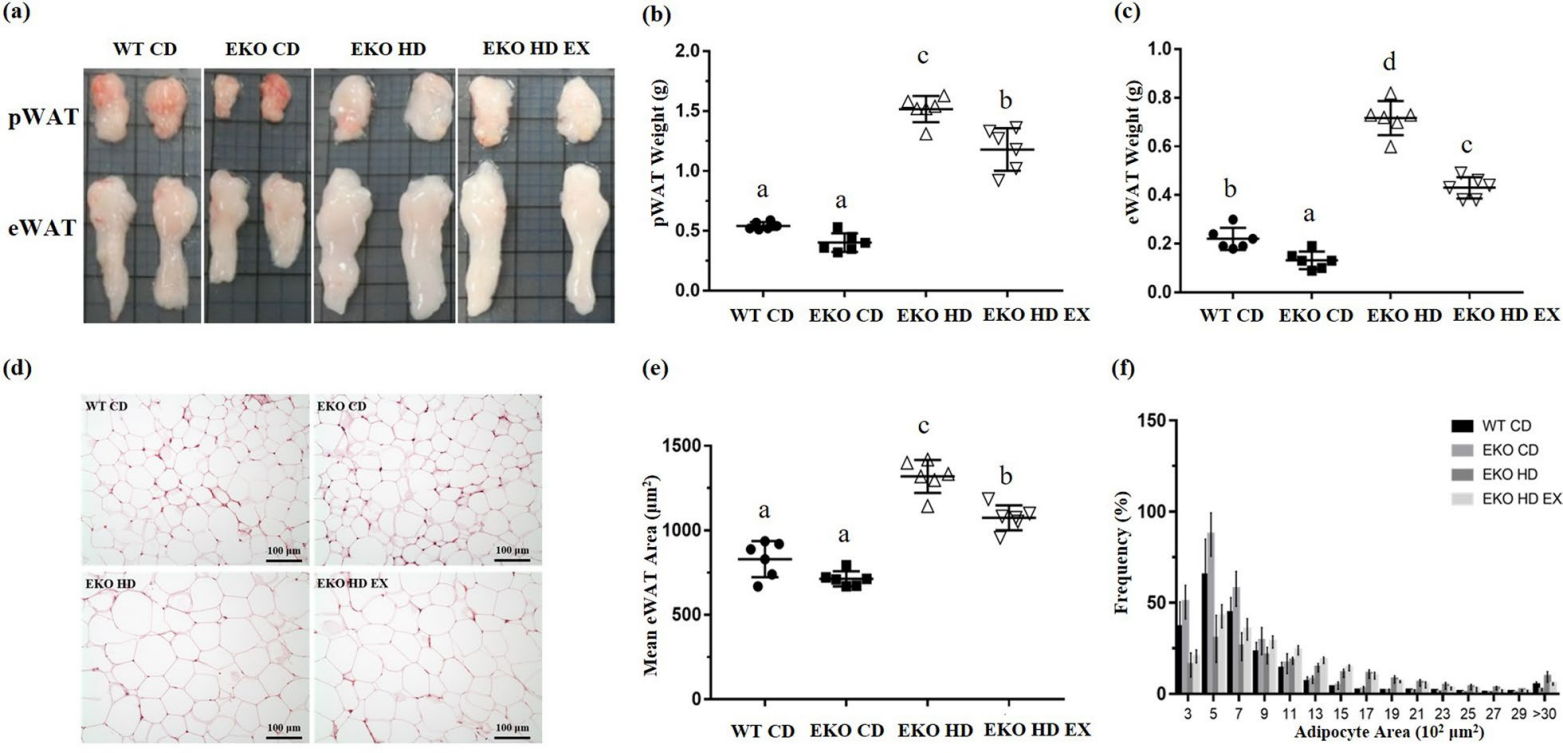
Effects of exercise on adipocyte morphology, weight, and distribution in *ApoE* mice fed a high-fat diet. Representative gross appearance of perirenal white adipose tissue (pWAT) and epididymal white adipose tissue (eWAT) (**a**). Fat mass of pWAT (**b**) and eWAT (**c**). Representative H&E staining of eWAT (**d**) to determine mean adipocyte size (**e**) and the distribution of adipocytes (**f**). Values are presented as the mean ± SD (*n* = 6). Different letters indicated significant differences among the groups (one-way ANOVA, *p* < 0.05). H&E, hematoxylin and eosin

The effects of exercise and the HFD intervention on the fat composition, pWAT, and eWAT are shown in Fig. 3. The total mass of pWAT and eWAT showed significant differences among the groups (F(3,20) = 132.9, *p* < 0.0001, and F(3,20) = 158.5, *p* < 0.0001, respectively). The mass of pWAT and eWAT was significantly higher in the EKO HD group versus the WT CD and EKO CD groups; and the exercise intervention markedly mitigated the HFD-induced adipose tissue hypertrophy in the EKO HD EX group compared with the EKO HD group (approximately 22% and 35%, respectively; Fig. 3b, c).

Obesity is characterized by an increase in the adipocyte area rather than an increase in the number of adipocytes. Figure 3d shows that hypertrophic adipocytes were observed in the HFD administration groups (EKO HD and EKO HD EX groups) compared with the normal CD groups (WT CD and EKO CD groups). In accordance with the area quantification, the mean eWAT adipocyte size also demonstrated significant differences among the groups (F(3,20) = 61.2, *p* < 0.0001). There was a significantly larger adipocyte area in the HFD groups (EKO HD and EKO HD EX groups) versus the normal CD groups (WT CD and EKO CD groups). The exercise intervention (i.e., in the EKO HD EX group) significantly decreased adipocyte hypertrophy by 18.5% compared with that noted in the EKO HD group (Fig. 3e). Moreover, the distribution of adipocytes exhibited a central tendency and there were higher proportions of adipocytes with areas of 500 μm^2^ in the normal CD groups (WT CD and EKO CD groups). The distribution in the HFD-fed groups shifted to the right, with a higher number of large adipocytes (> 3,000 μm^2^) observed (Fig. 3f). In the 12-week exercise training group (EKO HD EX group), adipocyte hypertrophy was alleviated with a notable increase in the fractions of smaller adipocytes (1,200 μm^2^ and 1,500 μm^2^) in comparison with the EKO HD group. These results demonstrated that exercise significantly mitigated the deleterious effects of HFD-induced higher fat composition and adipocyte hypertrophy.

### Effects of exercise on hepatic steatosis

**Fig. 4:**
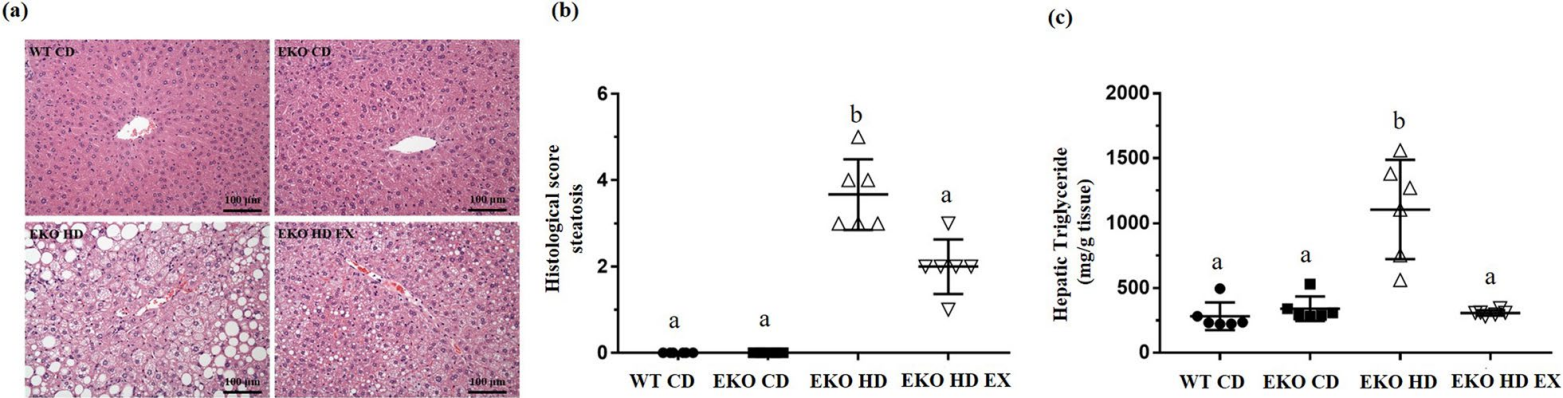
Effects of exercise on hepatic steatosis in *ApoE* mice feed fed a high-fat diet. Representative liver sections stained with H&E (**a**), the infiltration of lipid droplets into the hepatocytes with histological score (**b**) and the hepatic triglyceride contents (**c**) were evaluated. Values are presented as the mean ± SD (*n* = 6). Different letters indicate significant differences among the groups (one-way ANOVA, *p* < 0.05). H&E, hematoxylin and eosin

The hematoxylin and eosin staining of paraffin tissue sections collected from the four groups showed obvious hepatic steatosis in the HFD-induced groups (EKO HD and EKO HD EX groups). The exercise intervention led to milder pathological syndromes in the EKO HD EX group versus the EKO HD group (Fig. 4a). Regarding pathological scoring, there were significant differences in hepatic steatosis indices among the groups (F(3,20) = 80.7, *p* < 0.0001). The histological score in the EKO HD EX group was significantly decreased in comparison to that calculated for the EKO HD group. However, it remained significantly higher than those of the CD groups (Fig. 4b). Furthermore, changes in fats were also further quantified by determining the liver TG content using a colorimetric assay kit. The analysis revealed a significant difference in the hepatic TG content among the groups (F(3,20) = 22.9, *p* < 0.0001; Fig. 4c). The highest hepatic TG content levels were observed in the EKO HD group compared with the normal CD groups. In the exercise group (EKO HD EX group), the HFD-induced elevation in TG levels was significantly ameliorated in comparison with those recorded in the EKO HD group. Therefore, exercise could ameliorate HFD-induced accumulation of TGs in the liver and, consequently, alleviate the HFD-induced hepatic pathological syndromes.

### Multivariate analysis of the plasma lipidomic profile

**Fig. 5:**
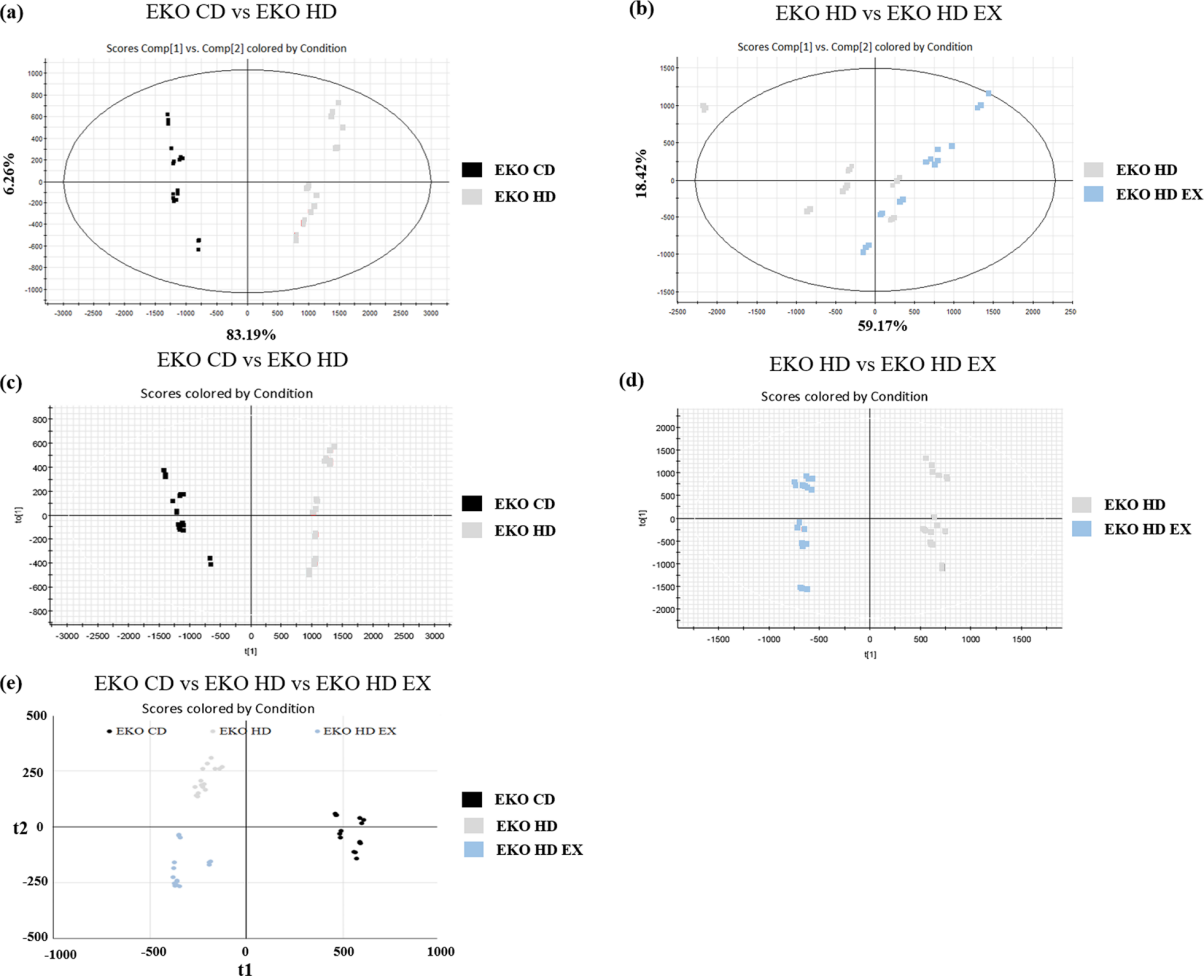
Multivariate analysis of the lipidomic profile after exercise and high-fat diet. Score plots from PCA and OPLS-DA. PCA score plot for the EKO CD versus EKO HD groups (**a**) and EKO HD versus EKO HD EX groups (**b**). OPLS-DA score plot for the EKO CD versus EKO HD groups (**c**), EKO HD versus EKO HD EX groups (**d**), and EKO CD versus EKO HD versus EKO HD EX groups (**e**). The x- and y-axes of PCA showing principal components 1 and 2, respectively, and the x- and y-axes of OPLS-DA showing the variance explained among and within the groups, respectively. OPLS-DA, orthogonal projections to latent structures discriminant analysis; PCA, principal component analysis

The plasmid lipidomic profiles of three groups were subjected to unsupervised and supervised multivariate analysis (PCA and OPLS-DA, respectively) to determine the possibility of distinguishing between the EKO CD, EKO HD, and EKO HD EX groups. The PCA score plot demonstrated clear clusters with significant differences for the EKO CD, EKO HD, and EKO HD groups (Fig. 5a, b). The PLS-DA score plot also revealed an obvious separation between the EKO CD, EKO HD, and EKO HD EX groups (Fig. 5c, d). As shown in Fig. 5e, the OPLS-DA results revealed that the plasma lipidomic profiles of the EKO CD, EKO HD, and EKO HD EX groups were clustered into three distinct groups. These findings indicated that HFD and exercise may affect lipid metabolism in mice. Based on validation through permutation tests, we obtained an R^2^ = 0.998 and Q^2^ = 0.907, which could be considered good compared to the standards for biological data (i.e., R^2^ = 0.7 and Q^2^ = 0.4).

### Effects of exercise and HFD on plasma lipids in ApoE knockout mice

**Fig. 6:**
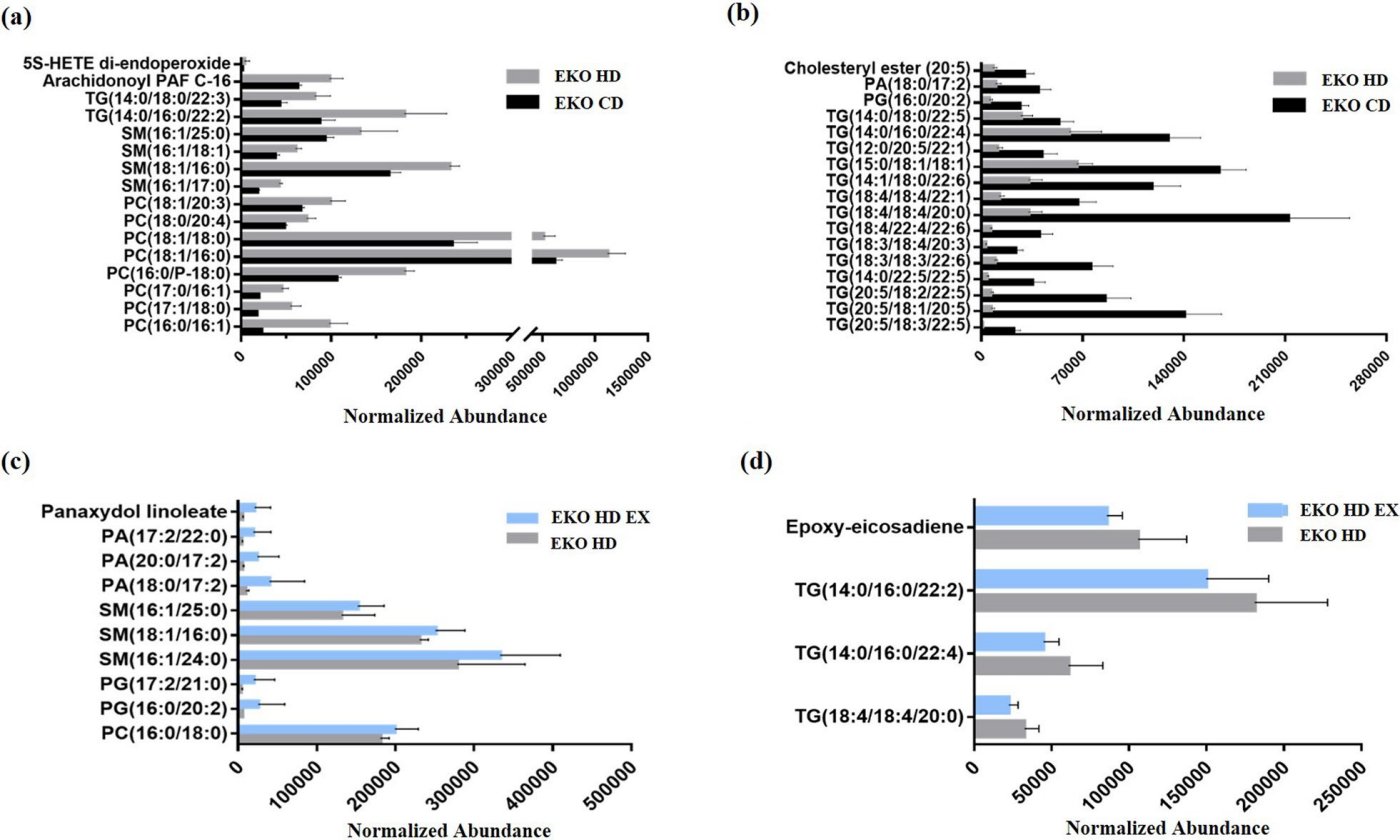
Effects of exercise and high-fat diet on lipid profiles. The difference between the EKO CD and EKO HD groups demonstrated significant decreases (**a**) and increases (**b**) in specific lipid species. Comparison of the EKO HD and EKO HD EX groups showed significant decreases (**c**) and increases (**d**) in specific lipid species. HETE, hydroxyeicosatetraenoic acid; PA, phosphatidic acid; PC, phosphatidylcholine; PG, phosphatidylglycerol; SM, sphingomyelin; TG, triglyceride

Based on the PCA and OPLS-DA results, plasma lipidomic profiles revealed obvious clusters, indicating the significance of different kinds of lipids between the EKO CD, EKO HD, and EKO HD groups. Significant increments in 16 species of lipids, mainly in phosphatidylcholine (PC; 16:0/16:1, 17:1/18:0, 17:0/16:1, 16:0/18:0, 18:1/16:0, 18:1/18:0, 18:0/20:4, and 18:1/20:3), sphingomyelin (SM; 16:1/17:0, 18:1/16:0, 16:1/18:1, and 16:1/25:0), TG (14:0/16:0/22:2, and 14:0/18:0/22:3), arachidonoyl PAF C-16, and HETE di-endoperoxide were observed in the EKO HD group compared with the EKO CD group (Fig. 6a). The levels of lipid species TG (20:5/18:3/22:5, 20:5/18:1/20:5, 20:5/18:2/22:5, 14:0/22:5/22:5, 18:3/18:3/22:6, 18:3/18:4/20:3, 18:4/22:4/22:6, 18:4/18:4/20:0, 18:4/18:4/22:1, 14:1/18:0/22:6, 15:5/18:1/18:1, 12:0/20:5/22:1, 14:0/16:0/22:4, and 14:0/18:0/22:5), phosphatidylglycerol (PG; 16:0/20:2), phosphatidic acid (PA; 18:0/17:2), and cholesteryl ester were significantly lower in the EKO HD group versus the EKO CD group (Fig. 6b). As shown in Fig. 6c, the exercise intervention after the administration of the HFD (in the EKO HD EX group) significantly elevated the levels of lipid species, mainly PC (16:0/18:0), PG (16:0/20:2, and 17:2/21:0), SM (16:1/24:0, 18:1/16:0, and 16:1/25:0), PA (18:0/17:2, 20:0/17:2, and 17:2/22:0), and panaxydol linoleate compared with those recorded for the EKO HD group. In addition, a significant reduction in lipid species, including TG (18:4/18:4/20:0), TG (14:0/16:0/22:4), TG (14:0/16:0/22:2), and epoxy-eicosadiene, was found in the EKO HD EX group versus the EKO HD group (Fig. 6d).

**Fig. 7:**
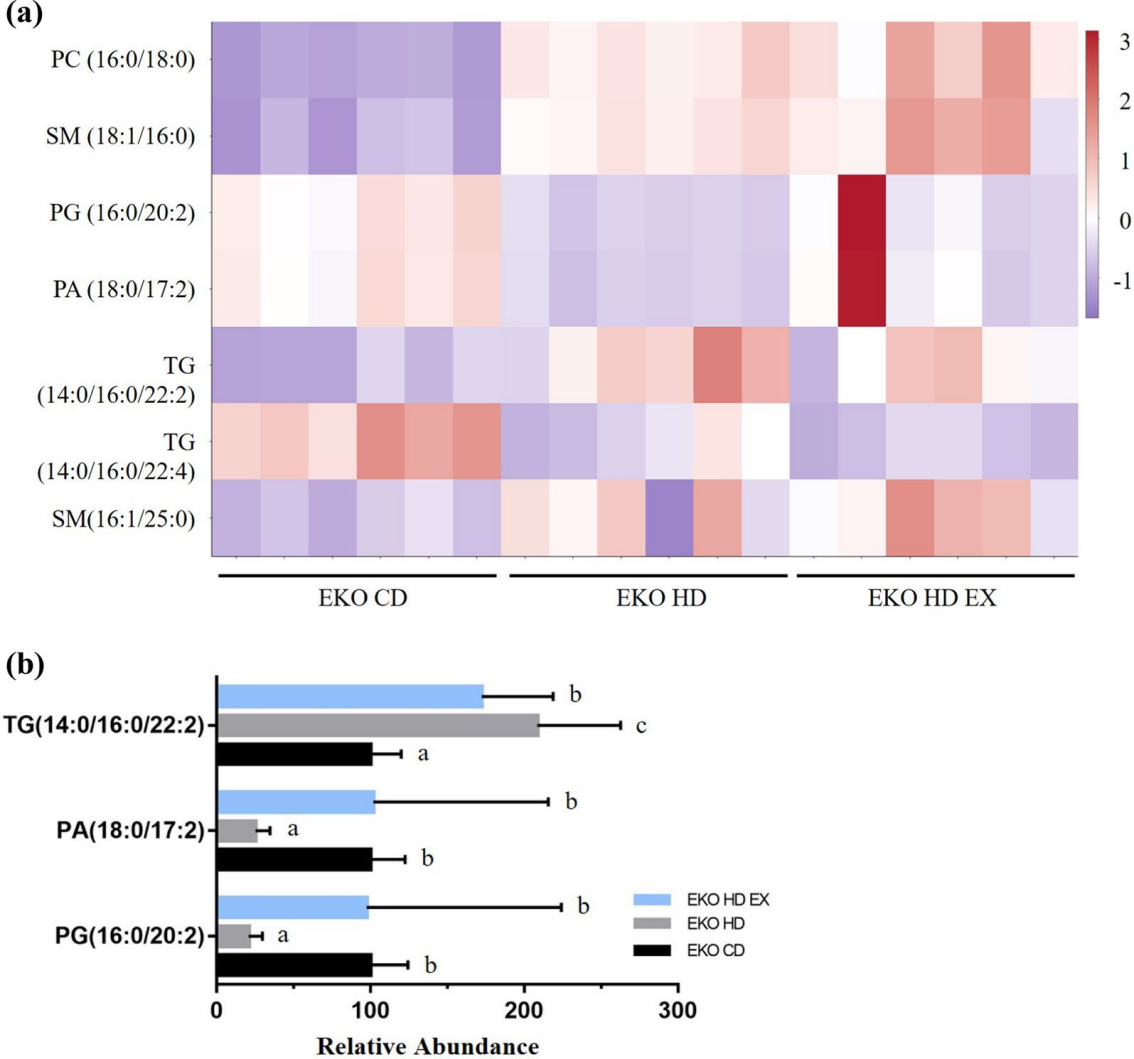
Correlation of specific lipid species with exercise and high-fat diet. Heatmap of integrated plasma lipid expression in the EKO CD, EKO HD, and EKO HD EX groups (**a**). The plasma lipid species, modulated by HFD, were reversed by the exercise intervention (**b**). The normalized values are presented using the following criteria: *p* < 0.05, FC > 1, and VI*p* > 1. Different alphabet (a, b) indicated significant differences among the groups (one-way ANOVA, *p* < 0.05). PA, phosphatidic acid; PC, phosphatidylcholine; PG, phosphatidylglycerol; SM, sphingomyelin; TG, triglyceride; FC, fold change; VIP, variable importance in the projection

According to the fluctuation in their levels, PC (16:0/18:0), SM (18:1/16:0; 16:1/25:0), PG (16:0/20:2), PA (18:0/17:2), and TG (14:0/16:0/22:2; 14:0/16:0/22:4) correlated and interacted with the effects of the HFD and exercise on the groups. The clusters of lipids exhibiting similar trends in expression among samples were converted using the Z-score scaling method for heatmap visualization (Fig. 7a). Taken together, the results showed that exercise significantly reversed the expression of three lipid species affected by HFD, namely TG (14:0/16:0/22:2), PA (18:0/17:2), and PG (16:0/20:2; Fig. 7b).

## Discussion

The high incidence of NAFLD is attributed to physical inactivity and excessive caloric intake. This is also strongly correlated with the rising trend of obesity and associated complications (e.g., cardiovascular diseases, diabetes, and metabolic diseases) posing great threats to public health. Regular and optimized exercise may offer multiple benefits in terms of cardiovascular fitness, systematic inflammation, oxidative stress, and weight control. Lipid profiles with physiological functions (e.g., membrane matrices, signaling, and energy depots) may be important modulators during the pathogenesis of NAFLD. Thus far, there are few studies investigating the beneficial effects of exercise on lipids in NAFLD. It has been shown that the *ApoE* knockout animal model with administration of HFD accelerates the progression and development of NAFLD because of hepatic inflammation, damage, and oxidized-LDL uptake sensitivity [20]. A 12-week exercise intervention in *ApoE* knockout mice that received a HFD significantly mitigated the induced obesity in terms of body weight gain, decrease of fat mass in body composition, and adipocyte morphology. In addition, the HFD-induced expression of plasma TG (14:0/16:0/22:2), PA (18:0/17:2), and PG (16:0/20:2) could be modulated by the exercise intervention to improve health.

*ApoE*, an important ligand of chylomicron remnants (very-low-density lipoprotein [VLDL] and HDL), binds to the hepatic receptor of LDL, VLDL and LDL receptor-related protein to facilitate the clearance of chylomicrons and VLDL remnants in plasma. An elevation of plasma cholesterol can be easily observed in *ApoE* knockout animal models. Furthermore, the *ApoE* knockout mouse model has been widely applied to mechanistic and therapeutic research on atherosclerosis [21]. In addition to atherosclerosis, the administration of a HFD to *ApoE* knockout mice induces the development of hyperlipidemia and accelerates the progression of NAFLD pathogenesis [22]. Excessive accumulation of lipids damages hepatocytes and leads to the elevation of the levels of liver-related enzymes in the serum (i.e., ALT and AST). This effect has also been observed in the biochemical parameters and hepatic steatosis in the present study. The pathogenesis of the *ApoE* knockout combined with a HFD also results in lipidosis and inflammation in lung tissue through the toll-like receptor 4 (TLR4) pathway [23]. Furthermore, Cao et al. also demonstrated an increase in ischemia susceptibility, inflammation, ganglion cell apoptosis, and retinal neovascularization in the *ApoE* knockout when combined with the administration of a HFD [24]. The accumulation of oxidized-LDL on tendons can decrease the functionality and induce a proliferative and matrix-degrading phenotype in tenocytes under *ApoE* knockout conditions after administration of a HFD [25]. Therefore, the *ApoE* knockout combined with HFD administration can exacerbate deleterious physiological effects and pathological developments in different types of tissues.

In previous work, obesity induced through the administration of a HFD significantly elevated the levels of TC, TG, LDL-c, and HDL-c compared with those recorded in normal controls and these increases could be ameliorated by a treadmill exercise intervention [26]. In a meta-analysis, aerobic exercise exerted beneficial effects on TC, TG, LDL-c, and HDL-c in patients with hyperlipidemia [27]. In our study, the increases in these indices induced by the administration of HFD were not affected by the endurance exercise intervention, although AST and ALT levels were affected. Therefore, the *ApoE* knockout may blunt the beneficial effects of exercise on TG, and cholesterol; nevertheless, exercise can still mitigate the steatosis-induced hepatic damage after the administration of a HFD. In animal studies involving the administration of a HFD and a methionine- and choline-deficient diet, elevated levels of AST and ALT could be observed in the NAFLD models with hepatic steatosis [28]. In agreement with previous findings using the *ApoE* knockout and HFD model, in the present study, the levels of AST and ALT were increased; this change was accompanied by hepatic TG accumulation and steatosis [29]. A healthy lifestyle drives the improvement in the levels of ALT, which are negatively associated with liver-related events and mortality, particularly in NAFLD [30]. The data demonstrated that endurance exercise significantly reduced the plasma levels of AST and ALT, indicating the amelioration of steatosis-induced hepatic injury.

The distribution and fluctuations in the levels of lipid species may be important modulators or biomarkers for the development of disease. It has been shown that PC (16:0/20:4) and SM (18:1/16:0) lipid species [31], which were induced in the present NAFLD model, are absent from the stem and terminal villi of human placentae with pathological malperfusion. Modulation of the same lipid species can be found in different types of diseases. The lipid species PC (18:1/22:6, 20:1/14:1, 20:3/20:4), PS (20:3/23:1), and PA (25:5/22:6) have also been identified as highly specific and sensitive biomarkers for the early diagnosis of endometriosis [32]. We found that the total TG content in the liver was significant higher in the EKO HD group compared to the EKO CD group. Nevertheless, only few TG (14:0/18:0/22:3 and 14:0/16:0/22:2) species in the plasma showed significantly higher levels in the NAFLD model (EKO HD group). Moreover, the majority of polyunsaturated TG lipid species were decreased in the NAFLD model group. This suggests that the lipid species may exhibit different profiles in the plasma and target tissues during the process of disease development. Therefore, the indicated lipid species may be useful biomarkers for disease surveillance. Furthermore, a recent study also reported a significant increment in PC and SM content in the plasma of patients with NAFLD and NASH versus healthy subjects [33]. In the present NAFLD model, the significant increase in the levels of PC and SM was consistent with previous results, and specific lipid species were also examined (Fig. 6a).

Metabolomics and lipidomics can be used to further examine the effects of aerobic/anaerobic or resistance exercise [34]. In cardiac disease, lipidomic profiling can also be used to validate the changes in lipid species (e.g., sphingolipid species and phospholipids containing omega-3/6 fatty acids) in response to swim endurance exercise [35]. In sarcoidosis, before and after the exercise intervention, the serum lipid profiles determined from spectrometer nuclear magnetic resonance spectra showed significant changes in the plasmalogen/TG ratio, cholesterol esters, and PC/SM. These are important determinants for the prevention of sarcoidosis complications [36]. Patients with NAFLD who exercised had significantly increased levels of specific sphingolipids and glycerophospholipids and reduced levels of metabolites (i.e., lipid X, 3-oxo hexacosanoic acid, epoxy-eicosene, and diglyceride) compared with NAFLD controls [37]. Following the exercise intervention in our study (Fig. 6c, d), we found that TG species (14:0/16:0/22:2, 14:0/16:0/22:4, and 18:4/18:4/20:0) and epoxy-eicosene showed a significant decrease, whereas specific PA, SM, and PG lipid species exhibited increases in the EKO HD EX group versus the EKO HD group. Collectively, the results showed that exercise could be considered a medical strategy with beneficial effects for the prevention and treatment of different types of disease. In the limitation of current study, the genetical background, the type of high fat diet, exercise types, and training duration could be variable factors to affect the NAFLD pathogenesis and alleviation. The ApoE model could be exacerbated with high fat diet induction model for acceleration of atherosclerosis and NAFLD. The pathological processes of NAFLD may be different from other induction model and clinic but the metabolism of lipid still could be possible similar for disease progress. Further investigation is warranted to analyze the profiles of related lipids affected by exercise. The changes in the fatty acid composition in NAFLD combined with exercise implied that specific lipid species levels may reflect improvement in the progression of NAFLD. Qualitative determination and structural analysis of all phospholipids should be performed to obtain metabolic insight into the pathogenesis of NAFLD from a lipidomics perspective. The present data of indicated lipid species could be possibly considered as potential biomarkers for clinic diagnosis, therapy, and improvement of NAFLD.

## Conclusions

Regular endurance exercise can ameliorate the pathogenesis of NAFLD caused by a HFD provided to *ApoE* knockout mice. The specific lipid species, including TG (14:0/16:0/22:2), PA (18:0/17:2), and PG (16:0/20:2), affected by an exercise intervention for NAFLD may also play critical roles in pathogenesis. To improve disease surveillance, the physiological functions of these lipids should be further assessed in future research.

## Data Availability

The data sets used and analyzed in this study are available from the corresponding author upon reasonable request.

## Abbreviations

NAFLD: Nonalcoholic fatty liver disease
HE: Haematoxylin and eosin
HFD: High-fat diet
TC: Total cholesterol
TG: Triglyceride
ALT: Alanine aminotransferase
AST: Aspartate aminotransferase
NASH: Non-alcoholic steatohepatitis
LDL-c: Low-density lipoprotein cholesterol
HDL-c: High-density lipoprotein cholesterol
MS: Mass spectrometry
ApoE: Apolipoprotein E
